# Early American Dentistry

**Published:** 1882-08

**Authors:** J. B. Hodgkin


### ARTICLE V.

### *Early American Dentistry.*

---

BY J. B. HODGKIN, D. D. S.

---

From an article in a Philadelphia newspaper, giving a history of the earlier days of that city, we condense some interesting items concerning the early days of dentistry of this country, and especially of Philadelphia. The practice of dentistry was at that time being taken from the hands of the barbers who had blended it with their calling, as a legitimate part of it. Of dentists, however, who made its practice exclusive, there were very few. One of the first who visited Philadelphia was a Dr. Lemayeur. This was nearly one hundred years ago—1784—and curiously the newly revived replantation and transplantation was in his practice a fashionable thing. He announced in an advertisement that he had successfully transplanted one hundred and twenty-three teeth in the preceding six months, and he offered two guineas for every tooth which might be offered him by persons disposed to sell their front teeth, or any of them. It would seem that the price of living teeth has not materially advanced, as a daily paper not long since contained the advertisement of a dentist offering two dollars—not so much by twenty-five cents, as was paid a hundred years ago.

The first resident dentist of Philadelphia who could claim something more than ordinary tooth-drawing skill, and who had scientific knowledge and experience, was Dr. Jacques Gardette, whose Christian name was soon Anglicised to James. He was a native of Agen, Department de Lot et Garonne, in France, where he was born on the 13th of August, 1756. He received an ordinary academical education in a provincial town. He was sent to Paris to study anatomy and surgery about 1773, and remained there two years as a student at the Royal Medical School. His tuition was considered to be completed after a course of eighteen months in the Hospital at Tours. After that he passed a regular examination at Bayonne by the surgeons of the admiralty, and received a commission as surgeon in the French navy. In the discharge of his official duty the vessel in which he served came to America in 1778, and shortly after his arrival Dr. Gardette resigned from the navy. He had previously received some instruction in dentistry in Paris, as a necessary part of his naval preparation, from M. Le Roy de la Faudiniere, a dentist in high repute, and had studied the only works in dentistry then extant by Fauchard and Bourdet. Dr. Gardette practiced in Boston and in Newport, and in 1783 established himself in New York. His success there not being very great he went to Philadelphia in the following year, and was engaged as a practitioner in dentistry for forty-five years afterward.

Dr. Gardette in 1785 resided in Pear Street, below Third. In 1791 he was at No. 88 Chestnut Street. In

1793 his office was at No. 75 Walnut Street, between Dock and Third, on the north side. This was the most fashionable part of the city. He removed to the corner of Ninth and Chestnut Streets in 1818. He retired from practice in 1829 and went to France, in order to revisit the scenes of his early life. He died at Bordeaux, in August, 1831. Gardette commenced the practice of dentistry at a time when the profession was but a few steps beyond the business of the tooth-drawer and the bleeder. Instruments were few, poorly made, awkward to handle, and needing forms and combinations which improvements in dental science have since introduced. Operations and processes were crude, and necessity must have compelled Gardette to introduce modifications and methods which have since become common professional property. He was the first to substitute elastic gold bands, or braces, in the place of ligatures of silk or fine gold wire in securing artificial teeth when attached to the living ones. He invented the manner of mounting natural teeth secured by means of gold pins upon a gold mortised plate, which permitted the teeth to rest upon the gum instead of upon the gold plate. He was the first to apply the principle of "suction," or atmospheric pressure, for the support of artificial teeth—doing away with the use of spiral springs and other inconvenient contrivances which filled up the mouth. This was done by him as early as 1800. The discovery of this principle was accidental, and was induced by finding that artificial teeth intended to be supplied with springs, but placed in the mouth of a lady in order to render her accustomed to them before the work was considered to be finished, were perfectly satisfactory to her without the springs when Dr. Gardette came to fix them, as he thought, permanently. Gold foil for filling teeth, instead of lead or tin, which was formerly used, was prepared by Gardette for his own use before the goldbeaters paid any attention to that manufacture. In 1822 the Franklin Institute awarded the Scott medal to Dr. Gardette for the three principal improvements in dentistry above mentioned ; also an Institute medal and a twenty-dollar prize for a lever instrument wlth which to extract teeth and stumps of teeth.

Dr. Edward Hudson was in practice in Philadelphia in 1802. He did not seem at first to find it congenial or pay ing, as we find him soon after in the stationery business and afterwards as a brewer. He resumed dentistry however in 1812, dying in 1833. The name of Hudson is familiar to those who have old dental books.

In September, 1808, Mr. Sanders, surgeon dentist, from London, announced that " his invented artificial teeth are of a composition of mineral harder than iron. Any color which never will change can be given to these teeth, and they can be made to fit so that no one can distinguish them from natural teeth. Mr. Sanders invites all persons whom accident, disease or age has occasioned to want elegant and useful aids to beauty, utterance and mastication, to honor him with a call. Decaying teeth can be preserved by filling their holes with a kind of cement which will stop the corruption, and prevent the toothache and bad smell which such teeth often occasion." Sanders did uot remain in the city very long. He was evidently not familiar with the " code," judging from his advertisement.

" L. Koecker, Surgeon Dentist, late from Europe," appeared in 1812. Since then the tables have turned and the European dentist advertises himtelf as " late from America." His " Principles of Dental Surgery," was declared by Harris to be " one of the best treatises that ever issued from the English press." Dr. Koecker returned to England about 1822, and afterward resided there. He published an essay on " Artificial Teeth, Obturators and Palates," in 1832, and an essay on " Diseases of the Jaw," in 1834.

The card of Josiah Drummond, in 1818, announces that he was " prepared by a regular course of experimental instruction, &c., to propose his services in all cases within the province of his profession, from the incipient forms of disease in dentition to the toothless defects of age or accident. Ladies and gentlemen who have objections to the use of foreign human teeth can be supplied with natural teeth which are not human to answer all the valuable purposes of the grafted human teeth." These "natural teeth, not human," were the teeth or preparations of the bones of animals.

About the same time, 1818, M. Dubuisson, announced that he had received "a beautiful collection of human teeth, which he offers to set on pivots only for $3, and $5 on springs." The era of cheap dentistry it seems set in quite a long time back, and advertising was freely made use of as a means of introducing the dentist, as is seen by the card of Dr. A. S. Van Pelt, in 1818, who gave notice that, "having gone through a regular course of study in the civil branches of his profession, and having reduced his theoretical knowledge to practice, tenders his services to his friends and the public as a dentist, &c. . . . He has engaged an Italian gentleman whose excellence, in an elegant and novel manner of inserting artificial, natural and human teeth, entitles him to rank among the most celebrated of the profession."

Just what "the civil branches of the profession" were in those days is matter for inquiry; but it is more than some dentists now-a-days succeed in, in "reducing their theoretical knowledge to practice." The Italian gentleman is introduced quite as handsomely as could be done in our day.

